# Second-generation antipsychotic use during pregnancy and risk of congenital malformations

**DOI:** 10.1007/s00228-021-03169-y

**Published:** 2021-06-08

**Authors:** Maria Ellfolk, Maarit K. Leinonen, Mika Gissler, Sonja Kiuru-Kuhlefelt, Leena Saastamoinen, Heli Malm

**Affiliations:** 1 Teratology Information, Department of Emergency Medicine Services, Helsinki University and Helsinki University Hospital, Tukholmankatu 17, 00029 HUS Helsinki, Finland; 2 Information Services Department, Data and Analytics, Finnish Institute for Health and Welfare, PB 30, 00271 Helsinki, Finland; 3 Information Services Department, Health and Social Services Data and Information Management Unit, Finnish Institute for Health and Welfare, PB 30, 00271 Helsinki, Finland; 4 Research Centre for Child Psychiatry, University of Turku, Lemminkäisenkatu 3, 20520 Turku, Finland; 5 Department of Molecular Medicine and Surgery, Karolinska Institute, 141 83 Huddinge, Sweden; 6 Research Unit, The Social Insurance Institution, Nordenskiöldinkatu 12, 00250 Helsinki, Finland; 7 Department of Clinical Pharmacology, Helsinki University and Helsinki University Hospital, PB 20 (Tukholmankatu 8 C), 00014 Helsinki, Finland; 8 Individualized Drug Therapy Research Program, Faculty of Medicine, University of Helsinki, PB 20 (Tukholmankatu 8 C), 00014 Helsinki, Finland

**Keywords:** Pregnancy, Second-generation antipsychotics, Major congenital malformations

## Abstract

**Purpose:**

To study if second-generation antipsychotic (S-GA) use during the first trimester of pregnancy is associated with an increased risk of major congenital malformations (MCM).

**Methods:**

A population-based birth cohort study using national register data extracted from the Drugs and Pregnancy database in Finland, years 1996–2017. The sampling frame included 1,273,987 pregnant women. We included singleton pregnancies ending in live or stillbirth or termination of pregnancy due to severe malformation. Pregnancies with exposure to known teratogens were excluded. Women were categorized into three groups: exposed to S-GAs (*n* = 3478), exposed to first-generation antipsychotics (F-GAs) (*n* = 1030), and unexposed (no purchases of S-GAs or F-GAs during pregnancy, *n* = 22,540). We excluded genetic conditions and compared the prevalence of MCMs in S-GA users to the two comparison groups using multiple logistic regression models.

**Results:**

Use of S-GAs during early pregnancy was not associated with an increased risk of overall MCMs compared to unexposed (adjusted odds ratio, OR 0.92; 95% CI 0.72–1.19) or to F-GA users (OR 0.82; 95% CI 0.56–1.20). Of individual S-GAs, olanzapine use was associated with an increased risk of overall MCMs (OR 2.12; 95% CI 1.19–3.76), and specifically, an increased risk of musculoskeletal malformations (OR 3.71; 95% CI 1.35–10.1) when compared to unexposed, while comparisons to F-GA users did not show significant results.

**Conclusions:**

Olanzapine use is associated with an increased risk of major congenital malformations and specifically, musculoskeletal malformations. Use during pregnancy should be restricted to situations where no safer alternatives exist.

**Supplementary information:**

The online version contains supplementary material available at 10.1007/s00228-021-03169-y.

## Introduction

Maternal well-being is important for a successful pregnancy outcome, and psychiatric illness must be treated adequately also during pregnancy. The clinician is often encountered by this dilemma, optimizing between effective maternal drug treatment and fetal safety. While antipsychotics are primarily used for psychotic illnesses, the second-generation antipsychotics (S-GAs) are also used in bipolar disorder for mood stabilization and in unipolar depression together with antidepressants [1]. Further, off-label use includes use in anxiety disorders, obsessive–compulsive disorders (OCD), post-traumatic stress disorder (PTSD) and insomnia [2].

The use of S-GA has been steadily increasing among pregnant women since year 2000; a recent study from ten countries reported that up to 2% of pregnant women use S-GAs, while there are differences across countries [3]. At the same time, the use of first generation antipsychotics (F-GA) has waned [3, 4]. The increasing use of S-GAs may be at least partly explained by the increasing off-label use of quetiapine for insomnia [2, 5].

Previous studies have not observed an association between F-GA or S-GA use and an increased risk of overall congenital malformations [6–8]. For the individual S-GAs, most data are available for aripiprazole, olanzapine, quetiapine and risperidone. Of these, only risperidone use has been associated with a small increased risk of congenital malformations and specifically, a marginally increased risk of congenital heart defects [6, 8]. While very few data are available for other organ specific malformations, no specific associations have been reported [9]. However, teratogens typically affect organ differentiation specifically, based on their pharmacodynamic and biological effects; an increased risk of organ specific malformations may therefore remain undetected when malformations are analyzed all together as a group [8, 10].

We evaluated the risk of overall major congenital malformations (MCM) and organ specific MCMs in offspring exposed to S-GAs in early pregnancy using national register data. Comparisons were made to unexposed and those exposed to F-GAs, controlling for maternal illness.

## Methods

### Data source and study cohort

This is a population-based birth cohort study using national register data extracted from the existing Drugs and Pregnancy database, established by the Finnish Institute for Health and Welfare (THL), the Social Insurance Institution of Finland (Kela), and the Finnish Medicines Agency (FIMEA). This database enables continuous surveillance of drug safety during pregnancy and includes data from the Medical Birth Register, the Abortion Register, the Register of Congenital Malformations, and the Prescription Register, including also the Special Refund Entitlement Register. Data from the different registers have been linked by the personal identification number assigned to all citizens and permanent residents in Finland. Data from births and terminations of pregnancy, and prescription drug purchases have been collected since January 1, 1996, and data in our study extend until December 31, 2017. In the database, beginning of pregnancy has been calculated from the best clinical estimation of gestational age at birth, primarily based on ultrasound. First trimester is defined as extending from the last menstrual period (LMP) until 84 days gestation.

More detailed information on the registers included have been published previously [4] and are also available in the Supplementary material.

### Definition of exposed and unexposed cohorts

**Fig. 1:**
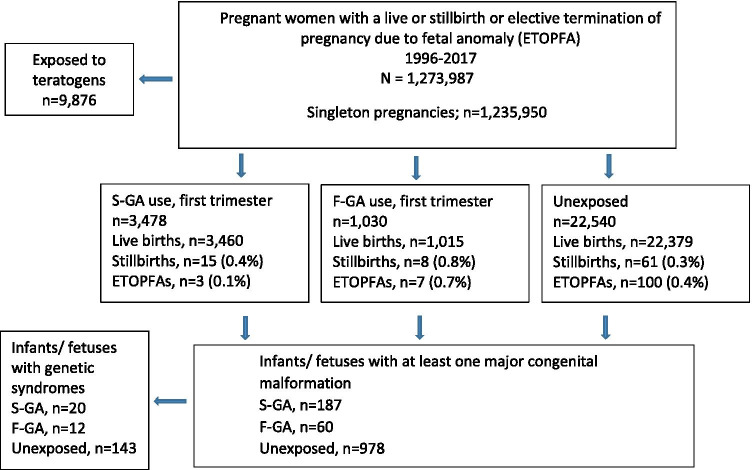
Flow chart of the exposure and outcome information used in the study

The study frame included 1,273,987 pregnancies ending in livebirth, stillbirth or elective termination of pregnancy due to fetal malformation (Fig. 1). We included 1,235,950 singleton pregnancies. Pregnancies exposed to known teratogens (Supplementary Table S1) during 3 months before pregnancy until end of pregnancy (*n* = 9876) were excluded from the study (Fig. 1).

#### Exposed to S-GAs

Women who purchased S-GAs (olanzapine, quetiapine, risperidone, aripiprazole, clozapine, ziprasidone, sertindol, or asenapine; Supplementary Table S2) during 1 month before pregnancy until the end of first trimester (*n* = 3478).

#### Unexposed

Women had no S-GA or F-GA purchases during three months before pregnancy until end of first trimester. Controls in this group were matched for year of birth of child and were randomly selected as five controls for one S-GA or F-GA exposed (5:1) (*n* = 22,540).

#### Exposed to F-GAs

Women who purchased F-GAs (Supplementary Table S2) during one month before pregnancy until the end of first trimester but did not purchase S-GAs during the same time period. Pregnancies exposed only to prochlorperazine, often used for morning sickness, (*n* = 392) were excluded. This comparison group was included to control for maternal psychiatric illness (*n* = 1030).

### Major congenital malformations

The outcomes of interest were overall MCMs, i.e., infant/fetus with at least one major congenital malformation and organ-specific malformations, including cardiovascular (ICD-9 diagnoses 745–747), central nervous system (ICD-9; 740–742) respiratory tract malformations (ICD-9; 748), orofacial clefts (ICD-9; 7490–7492), urogenital (ICD-9; 752,753), gastrointestinal malformations (ICD-9; 750,751, 7566), and musculoskeletal malformations (ICD-9; 7543–7548, 755). We excluded genetic conditions from the analyses (Fig. 1).

### Covariates

Covariates included maternal sociodemographic and medical characteristics and use of other medication, categorized as shown in Supplementary Table S3. Data on pre-pregnancy BMI are partially available beginning from 2004 and for all women from September 2005. Alcohol use is not routinely collected in the MBR and could therefore not be included in analyses.

### Statistical analyses

All data in the Drugs and Pregnancy database are pseudonymized. We made a descriptive analysis on demographic differences between study cohorts. We also calculated the prevalence of overall MCMs and organ-specific malformations in the S-GA group, the unexposed group, and the F-GA group, and further, on the level of individual S-GAs.

We used logistic regression to assess the association between S-GA use during preconception or first trimester and MCMs in comparison with unexposed and F-GA exposed pregnancies. First, pattern of missingness for socioeconomic status, smoking, cohabitation pre-pregnancy BMI, and parity was explored as appropriate. The majority of missingness was driven in one missing value either in socio-economic status or BMI. Incomplete records were assumed to be missing at random, and we used multiple imputation by chained equations (MICEs) to predict missing values in covariates to improve measurement of prognostic factors [11, 12]. Imputation models included outcome variables, exposure variables, covariates and also auxiliary variables that correlated or were believed to be associated with missingness (birth weight, gestational age, number of drug purchases and hospital district). We created 20 imputed data sets of which estimates were combined using Rubin’s rules [13, 14].

The crude models included adjustment for year of delivery. For the adjusted analyses, clinically relevant and plausible covariates were first tested for association with the three-class exposure status. When associated with exposure at significance level *P* < 0.1, the covariate was further tested separately for association of outcome. We included in the final logistic regression models covariates which were associated with exposure and outcome at *P* < 0.1 as potential confounders (Supplementary Table S4). An infant/fetus with multiple organ-specific MCMs contributed to each malformation subgroup. All analyses were performed using SAS Enterprise Guide 7.1(SAS Institute Inc., Cary, NC, USA).

The utilization of sensitive health register data for scientific research and the data linkages in the Drugs and Pregnancy project have been approved by the register administrators and the national data protection authority. Since the study subjects are not contacted, according to the Finnish legislation informed consent is not required. The study was registered in The European Network of Centres for Pharmacoepidemiology and Pharmacovigilance (ENCePP) register before data collection started (EUPAS4799). The study has been granted the ENCePP seal, following the ENCePP principles of standards, transparency and independence of good pharmacoepidemiology practice throughout the research process (www.encepp.eu).

## Results

Overall, 4508 (0.4%) pregnant women with a singleton pregnancy (*N* = 1,235,950) used antipsychotics during the first trimester or 30 days before pregnancy, and 3478 (0.3%) used S-GAs. The number of stillbirths and ETOPFAs were small across the exposure groups (Fig. 1).

Maternal characteristics of the three study groups are presented in Supplementary Table S3. S-GA users were more likely to be overweight than F-GA users or unexposed and S-GA and F-GA users were more likely to smoke tobacco than women in the unexposed group. Pre-gestational diabetes and gestational diabetes were significantly more common among the S-GA users compared to F-GA users and to the unexposed group.

The numbers of excluded infants/fetuses with genetic conditions are presented in Fig. 1. Of the individual S-GAs, the most commonly used was quetiapine (*n* = 2618), followed by olanzapine (*n* = 413), risperidone (*n* = 242), aripiprazole (*n* = 220), and clozapine (*n* = 106).

The prevalence and risk of overall MCMs are presented in Tables 1 and 2. Compared to unexposed, the risk did not differ between any S-GA users and unexposed (Table 1). Of the individual S-GAs, olanzapine use was associated with a twofold increased risk of MCMs (odds ratio, OR 2.12; 95% CI 1.19–3.76) after full adjustment to confounders.

**Table 1:** Prevalence and risk of overall major congenital malformations in pregnancies of second-generation antipsychotic users. Comparisons to the unexposed

Exposure	Prevalence 1/1000	Crude OR*	95% CI	Adjusted** OR	95% CI
Unexposed	43.7	Ref	Ref	Ref	Ref
Any S-GA	54.1	**1.20**	**1.02–1.41**	0.92	0.72–1.19
Quetiapine	49.3	1.07	0.88–1.29	0.85	0.64–1.12
Olanzapine	79.9	**1.90**	**1.33–2.73**	**2.12**	**1.19–3.76**
Risperidone	53.7	1.29	0.73–2.26	1.48	0.75–2.90
Aripiprazole	72.7	1.60	0.96–2.68	1.80	0.74–4.42
Clozapine	37.7	0.88	0.32–2.41	2.00	0.30–13.35

Analyses based on imputed data

*S-GA* second-generation antipsychotic

^*^Adjusted for year of delivery; ^**^Adjusted for year of delivery, maternal age at delivery, parity, pre-pregnancy BMI, cohabitation, smoking, SES, other psychiatric drugs, psychotic and other severe mental disorders, pre-gestational diabetes, gestational diabetes

Statistically significant results written in bold

**Table 2:** Prevalence and risk of overall major congenital malformations in pregnancies of second-generation antipsychotic users. Comparisons to first-generation antipsychotic users

Exposure	Prevalence 1/1000	Crude OR*	95% CI	Adjusted** OR	95% CI
Any F-GA	58.9	Ref	Ref	Ref	Ref
Any S-GA	54.1	0.88	0.60–1.29	0.82	0.56–1.20
Quetiapine	49.3	0.80	0.52–1.24	0.76	0.49–1.18
Olanzapine	79.9	1.40	0.84–2.32	1.33	0.78–2.27
Risperidone	53.7	0.89	0.47–1.71	0.83	0.42–1.61
Aripiprazole	72.7	1.38	0.68–2.79	1.06	0.50–2.24
Clozapine	37.7	0.67	0.23–1.92	0.51	0.16–1.57

Analyses based on imputed data

*S-GA* second-generation antipsychotic, *F-GA* first-generation antipsychotic

^*^Adjusted for year of delivery; ^**^Adjusted for year of delivery, maternal age at delivery, parity, pre-pregnancy BMI, cohabitation, smoking, SES, other psychiatric drugs, psychotic and other severe mental disorders, pre-gestational diabetes, gestational diabetes

The risk of MCMs in any S-GA users was lower than in the F-GA group; however, this difference was not statistically significant (Table 2). Of the individual S-GAs, olanzapine use was associated with an increased risk of any MCM when compared to F-GA users, but did not reach statistical significance.

Use of any S-GA was not associated with an increased risk of any organ specific malformations when compared to unexposed or to the F-GA group (Tables 3 and 4). Of the individual S-GAs, olanzapine use was associated with a nearly fourfold increased risk of musculoskeletal malformations when compared to unexposed (OR 3.71; 95% CI 1.35–10.1). Compared to F-GA users, the risk of musculoskeletal malformations was higher but the association did not remain statistically significant (Table 4). The musculoskeletal malformations recorded among the olanzapine exposed infants/fetuses were various and no pattern of malformations was observed.

**Table 3:** Organ system specific major congenital malformations in pregnancies of second-generation antipsychotic users. Comparisons to unexposed

Exposure	Cardiovascular		Central nervous system		Respiratory tract		Orofacial cleft		Gastrointestinal		Urogenital		Musculoskeletal	
	Prev 1/1000	Adj. OR (95% CI)	Prev 1/1000	Adj. OR (95% CI)	Prev 1/1000	Adj. OR (95% CI)	Prev 1/1000	Adj. OR (95% CI)	Prev 1/1000	Adj. OR (95% CI)	Prev 1/1000	Adj. OR (95% CI)	Prev 1/1000	Adj. OR (95% CI)
Unexposed	16.9	Ref	2.1	Ref	0.9	Ref	1.5	Ref	2.0	Ref	5.7	Ref	10.4	Ref
Any S-GA	22.0	0.92 (0.62–1.36)	2.3	0.44 (0.13–1.53)	0.3	0.12 (0.01–2.11)	1.7	0.36 (0.07–1.80)	2.0	0.37 (0.09–1.45)	7.8	0.88 (0.45–1.72)	12.4	0.95 (0.58–1.57)
Quetiapine	20.6	0.94 (0.61–1.43)	2.3	0.53 (0.15–1.91)	0.0	NA	0.8	0.20 (0.02–1.64)	0.8	0.16 (0.02–1.11)	7.6	0.93 (0.45–1.88)	10.3	0.67 (0.38–1.19)
Olanzapine	31.5	1.83 (0.74–4.52)	4.8	1.00 (0.08–12.6)	2.4	1.43 (0.04–46.2)	4.8	1.25 (0.07–21.6)	4.8	4.33 (0.63–29.6)	9.7	2.88 (0.71–11.6)	19.4	**3.71 (1.35–10.1)**
Risperidone	12.4	0.74 (0.20–2.78)	4.1	1.00 (0.06–15.7)	0.0	NA	4.1	4.76 (0.48–47.1)	4.1	1.19 (0.08–18.0)	8.3	1.23 (0.19–7.89)	16.5	2.77 (0.92–8.29)
Aripiprazole	50.0	1.94 (0.58–6.53)	0.0	NA	0.0	NA	4.5	3.69 (0.06–216.4)	4.5	1.96 (0.04–94.5)	9.1	4.35 (0.62–30.6)	13.6	1.21 (0.19–7.77)

Analyses based on imputed data

*S-GA* second-generation antipsychotic, *Prev* prevalence, *Adj. OR* adjusted odds ratio adjusted for year of delivery and covariates associated with exposure outcome at *P* < 0.1

Statistically significant results written in bold

**Table 4:** Organ system specific major congenital malformations in pregnancies of second-generation antipsychotic users. Comparisons to first-generation antipsychotic users

Exposure	Cardiovascular		Central nervous system		Respiratory tract		Orofacial cleft		Gastrointestinal		Urogenital		Musculoskeletal	
	Prev 1/1000	Adj. OR (95% CI)	Prev 1/1000	Adj. OR (95% CI)	Prev 1/1000	Adj. OR (95% CI)	Prev 1/1000	Adj. OR (95% CI)	Prev 1/1000	Adj. OR (95% CI)	Prev 1/1000	Adj. OR (95% CI)	Prev 1/1000	Adj. OR (95% CI)
Any F-GA	21.6	Ref	6.9	Ref	2.0	Ref	2.0	Ref	2.9	Ref	7.9	Ref	19.6	Ref
Any S-GA	22.0	0.80 (0.44–1.44)	2.3	0.53 (0.12–2.29)	0.3	0.15 (0.00–5.85)	1.7	0.67 (0.09–4.78)	2.0	0.70 (0.12–3.98)	7.8	0.62 (0.24–1.60)	12.4	0.67 (0.32–1.38)
Quetiapine	21.6	0.74 (0.38–1.44)	2.3	0.36 (0.07–1.76)	0.0	NA	0.8	0.48 (0.03–6.92)	0.8	0.20 (0.02–2.28)	7.6	0.60 (0.21–1.66)	10.3	0.59 (0.24–1.42)
Olanzapine	31.5	0.95 (0.41–2.16)	4.8	0.98 (0.15–6.49)	2.4	1.91 (0.04–83.7)	4.8	2.05 (0.17–24.7)	4.8	2.41 (0.24–24.6)	9.7	0.95 (0.23–3.89)	19.4	1.30 (0.46–3.66)
Risperidone	12.4	0.46 (0.13–1.66)	4.1	1.09 (0.11–11.3)	0.0	NA	4.1	0.55 (0.01–20.4)	4.1	1.31 (0.10–16.6)	8.3	0.58 (0.11–3.20)	16.5	0.76 (0.23–2.48)
Aripiprazole	50.0	1.33 (0.50–3.54)	0.0	NA	0.0	NA	4.5	0.47 (0.01–29.0)	4.5	3.03 (0.12–76.0)	9.1	0.54 (0.08–3.58)	13.6	0.57 (0.12–2.73)

Analyses based on imputed data

*S-GA* second-generation antipsychotic, *F-GA* first-generation antipsychotic, *Prev* prevalence, *Adj. OR* adjusted odds ratio adjusted for year of delivery and covariates associated with exposure and outcome at *P* < 0.1

## Discussion

In this study based on national register data, use of second-generation antipsychotics during early pregnancy was not associated with an increased risk of overall major congenital malformations compared to unexposed pregnancies or to pregnancies where the woman used first-generation antipsychotics. Of the individual S-GAs, olanzapine use was associated with a twofold increased risk of overall malformations, and specifically, a nearly fourfold increased risk of musculoskeletal malformations when compared to unexposed.

Use of S-GAs has been increasing among the pregnant population [3, 4] and indications for S-GA use have expanded during the last decade to include also illnesses outside psychotic disorders [3, 5]. S-GAs are used increasingly in treating bipolar disorder and as augmentation treatment in unipolar depression [15]. Further, S-GAs may be preferred during pregnancy because of safety concerns related to antiepileptic drugs as mood stabilizers [3]. Antipsychotic augmentation may also be effective in treatment-refractory OCD [16]. Present guidelines state that there is insufficient evidence to recommend for or against antipsychotic medication for PTSD [17] and use should be restricted to situations where symptoms have not responded to other drug or psychological treatments [18]. However, a European study reported that nearly 60% of more than 1,000 patients with a diagnosis of PTSD were prescribed antipsychotics, most commonly quetiapine, olanzapine and risperidone [19]. Further, a population-based study from Norway suggested that the majority of quetiapine prescriptions during 2004–2015 were for indications other than psychosis [5]. S-GAs and particularly quetiapine are used off-label for anxiety disorders and sleep disturbances with little evidence to support use for insomnia [20, 21]. There are no data about S-GA use during pregnancy and specific indications.

Our finding that S-GAs as a group do not increase the risk of congenital malformations is in line with most previous studies. A prospective follow-up study including more than 500 pregnancies following first trimester exposure to S-GAs and including also pregnancy terminations reported an increased risk of overall malformations in S-GA users when compared to unexposed (OR, 2.13; 95% CI, 1.19–3.83) but the risk was mainly attributed to cardiac septal defects, and the authors concluded that the results likely resulted from detection bias [9]. No increased risk of congenital malformations was observed in a cohort study based on health administrative databases in Canada and including approximately 1,000 S-GA exposed pregnancies ending in birth, applying high dimensional propensity score matching analyses [7]. Also, the largest study based on Medicaid data including more than 9,000 pregnancies exposed to S-GAs and ending in live birth did not observe an increased risk of overall (OR 1.05; 95% CI 0.96–1.16) or cardiac (OR 1.06; 95% CI 0.90–1.24) malformations in the fully adjusted, propensity score-based analysis. Of the individual drugs, only risperidone use (> 1500 pregnancies) was associated with an increased risk of overall malformations (OR 1.26; 95% CI 1.02–1.56) and a marginally significant risk of cardiac malformations (OR 1.26; 95% CI 0.88–1.81) [6]. No sign of teratogenicity has been reported for aripiprazole, quetiapine, or olanzapine; the number of reported olanzapine exposed pregnancies analyzed for malformations totals close to 3,000 pregnancies [6, 8, 22]. Contrary to these findings, olanzapine use in our study was associated with a substantially increased risk of overall malformations and specifically with musculoskeletal malformations when compared to the unexposed cohort.

The association of olanzapine use with an increased risk of overall malformations was unexpected, given the reassuring data from the previous studies [6–8]. Very few data exist for other specific malformations than congenital cardiac defects. It is possible that an increased risk of organ specific malformations (other than cardiac defects) may have remained undetected in studies which have focused on the overall malformation risk. Olanzapine has not been teratogenic in animal studies.

One study based on prospectively collected data in relation to outcome reported no major differences in organ specific malformations, including musculoskeletal malformations in S-GA users compared to F-GA users or to pregnancies with no exposure but the total number of S-GA exposed pregnancies available for analyses was only 430, and no results were reported for individual S-GAs [9]. S-GA treatment may cause disturbances in glucose metabolism, leading to hyperglycemia and diabetes, and poor glycemic control before pregnancy and in early pregnancy is associated with an increased risk of congenital malformations [23, 24]. Typical malformations related to maternal hyperglycemia include cardiovascular and central nervous system malformations, but also musculoskeletal malformations [23, 25]. While impaired glucose metabolism is a common side effect of all S-GAs, one would expect to find an increased malformation risk associated with any S-GA use and also with each individual S-GA. However, olanzapine of the S-GA drug group has the highest diabetogenic properties [26] and could therefore differ from the other S-GAs in its teratogenic potential. If our findings were related to maternal hyperglycemia, it is difficult to explain why previous studies have not shown an association between olanzapine use and an increased risk of overall congenital malformations or cardiac malformations. Maternal hyperglycemia is also an unlikely explanation, as we adjusted for pregestational and gestational diabetes in the analyses for overall malformations, and for pregestational diabetes in the analyses of organ specific malformations,

The discrepant findings compared to previous research might even be related to genetic differences in drug metabolizing enzyme activity across populations. Olanzapine is metabolized by hepatic glucuronidation and by cytochrome P450 (CYP) 1A2 and to a minor extent by CYP 2D6 -mediated oxidation to inactive metabolites. Ethnic differences exist for both CYP enzymes but CYP 1A2 activity does not differ significantly between the Finnish population and other Caucasians populations [27]. Further, the findings related to the impact of CYP1A2 and UDP-glucuronosyltransferase (UGT) 1A4 variants on olanzapine steady-state concentrations have been conflicting [28, 29]. The genetic profile of CYP2D6 in the Finnish population differs from North European populations in that the frequency of the ultrarapid metabolizer genotype is higher and that of poor metabolizer is lower in the Finnish population [30]. However, CYP2D6 polymorphism appears to have no significant influence on olanzapine pharmacokinetics [28, 29]. No further conclusions related to pharmacogenetic differences can be made as we had no genetic information on the subjects.

The risk of overall and musculoskeletal malformations in olanzapine exposed pregnancies was not statistically significantly increased when compared to F-GA users, suggesting that maternal illness or illness-related factors may have contributed to the findings. However, the risk estimates for olanzapine in these comparisons—even if not statistically significant -were strikingly different from the other S-GAs, showing a risk increase of round 30% for both overall and musculoskeletal malformations, while the risk estimates for other S-GAs were below or close to one.

Our study has several strengths. First, we included a comparison group of women exposed to F-GAs, controlling for maternal illness. Second—and contrary to previous studies—we included pregnancy terminations due to fetal malformation, which is important as more than 10% of all pregnancies where the fetus has been diagnosed with a major malformation are currently terminated [31]. Third, the reimbursement register includes 99% of all reimbursed medications [32], and the National Medical Birth Register data have been validated and are considered good [33]. Further, the data sources included in our study allowed for extensive control of potential confounders.

The limitations are those typical for studies using large administrative databases and national register data. Exposure misclassification may occur if the woman does not take her medication even if she bought it in the pharmacy, moving the risk estimate towards one if the drug was a teratogen. However, antipsychotic drugs are generally prescribed for psychotic conditions needing drug treatment and their use continues in a relatively constant manner from preconception through first trimester [34]. Residual confounding is an unlikely explanation to our findings as one would expect residual confounding to affect the results of all individual S-GAs and not only olanzapine.

We conclude that olanzapine use in early pregnancy is associated with an increased risk of overall malformations, and specifically musculoskeletal malformations. Until these results are either confirmed or refuted, use of olanzapine should only be used in pregnancy when no safer alternatives exist.

## Supplementary Information

Below is the link to the electronic supplementary material.

Supplementary file1 (DOCX 26 KB)

## Data Availability

The data that support the findings of this study are not publicly available. According to the national data protection legislation, permission to obtain the research data must be applied from the Finnish Institution for Health and Welfare.
