# Supplementary material for: Second-generation antipsychotic use during pregnancy and risk of congenital malformations
Source: Eur J Clin Pharmacol. 2021 Jun 8;77(11):1737–45. doi: 10.1007/s00228-021-03169-y (PMC8528770; doi:10.1007/s00228-021-03169-y)
Supplement: Supplementary file 1 — Supplementary file1 (DOCX 26 KB) [file 228_2021_3169_MOESM1_ESM.docx]

**Supplementary material.**

| **Description of registers** | 2 |
| --- | --- |
| **Supplementary Tables** |  |
| **Table S1.** Known teratogens removed from study material before extraction of exposure groups. | 3 |
| **Table S2**. First- and second-generation antipsychotics included in the study. | 4 |
| **Table S3.** Maternal characteristics. | 5 |
| **Table S4.** Maternal characteristics tested as covariates by study outcome in imputed data. | 7 |
|  |  |

**Description of the registers included in the study.**

*The Medical Birth Register (MBR)* maintained since 1987 by THL is a nationwide register collecting data on maternal demographic characteristics, medical and reproductive history, and neonatal outcome data up to six days’ age. The register data include all live births and stillbirths with gestational age of 22 weeks or more or birth weight of 500 grams or more, and the completeness of births is close to 100%. The definitions and variables included in this registry are based on established international concepts and use the 10^th^ version of the WHO International Statistical Classification of Diseases and Related Health Conditions (ICD) since 1996 (children) and 2004 (mothers).

*The Register of Congenital Malformations*, established in 1963 and maintained by THL, contains national-level data on congenital chromosomal and structural malformations detected or suspected in stillborn and live born infants and fetuses. Data are collected from several sources including hospitals, health care professionals and other national registers including the MBR. The register uses the extended ICD-9 coding since 1986 (Congenital anomalies 2017 ) [[10](#_ENREF_10)] and collects primarily data on MCM using the European network of population-based registries for the epidemiological surveillance of congenital anomalies (EUROCAT) criteria for exclusion ( <https://eu-rd-platform.jrc.ec.europa.eu/eurocat_en>). The register contains also basic information about maternal background data.

*The Finnish Prescription Register* (Kela) contains data on reimbursed prescription drug purchases. Prescription-only medicines deemed necessary for treatment of an illness are reimbursed under the national social insurance covering all permanent residents in Finland. Drug purchases are reimbursed concomitantly with the purchase at pharmacies and drugs are supplied to the patient for a maximum of three months at a time. Data in the register include the date of purchase and the International Anatomic-Therapeutic-Chemical (ATC) classification code (<https://www.who.int/classifications/atcddd/en/>). Over-the-counter drugs or medications given to institutionalized persons are not included in the register. The Kela also maintains the Special Refund Entitlement Register since 1964 with data on patients who are entitled for higher reimbursement for chronic illnesses requiring continuous drug treatment. These illness and treatment codes also include severe psychiatric conditions.

**Supplementary Table S1.** Known teratogens removed from study material before extraction of exposure groups.

| **Drug** | **ATC-code** |
| --- | --- |
| Misoprostol | A02BB01, M01AB55 |
| Warfarin | B01AA03 |
| Agents acting on the renin-angiotensin system | C09 |
| Etretinate | D05BB01 |
| Acitretin | D05BB02 |
| Isotretinoin | D10BA01 |
| Alitretinoin | D11AH04 |
| Carbimazole | H03BB01 |
| Antineoplastic agents | L01 |
| Mycophenolic acid | L04AA06 |
| Leflunomide | L04AA13 |
| Teriflunomide | L04AA31 |
| Thalidomide | L04AX02 |
| Methotrexate | L04AX03 |
| Lenalidomide | L04AX04 |
| Pomalidomide | L04AX06 |
| Ergot alkaloids | N02CA |
| Lithium | N05AN01 |
| Valproic acid | N03AG01 |
| Carbamazepine | N03AF01 |
| Phenytoin and its derivates | N03AB |
| Topiramate | N03AX11 |

**Supplementary Table S2**. First- and second-generation antipsychotics included in the study.

| **First generation antipsychotics (F-GA)** | |
| --- | --- |
| **ATC code** | **Generic name** |
| N05AA01 | Chlorpromazine |
| N05AA02 | Levomepromazine |
| N05AB01 | Dixyrazine |
| N05AB02 | Fluphenazine |
| N05AB03 | Perphenazine |
| N05AB04 | Prochlorperazine |
| N05AC01 | Periciazine |
| N05AC02 | Thioridazine |
| N05AD01 | Haloperidol |
| N05AD03 | Melperone |
| N05AF01 | Flupentixol |
| N05AF03 | Chlorprotixen |
| N05AF05 | Zuclopentixol |
| N05AL01 | Sulpiride |
| **Second generation antipsychotics (S-GA)** | |
| **ATC code** | **Generic name** |
| N05AE03 | Sertindol |
| N05AE04 | Ziprasidone |
| N05AE05 | Lurasidone |
| N05AH02 | Clozapine |
| N05AH03 | Olanzapine |
| N05AH04 | Quetiapine |
| N05AH05 | Asenapine |
| N05AL02 | Sultopride |
| N05AL04 | Remoxipride |
| N05AL05 | Amisulpride |
| N05AL07 | Levosulpiride |
| N05AX08 | Risperidone |
| N05AX10 | Mosapramine |
| N05AX11 | Zotepine |
| N05AX12 | Aripiprazole |
| N05AX13 | Paliperidone |
| N03AX14 | Iloperidone |
| N05AX15 | Cariprazine |
| N05AX16 | Brexpiprazole |

**Supplementary Table S3.** Maternal characteristics.

|  | **Second-generation antipsychotics**  **(S-GAs)** | | **First-generation**  **antipsychotics**  **(F-GAs)** | | **Unexposed** | | **Covariate**  **associated**  **with**  **exposure** |
| --- | --- | --- | --- | --- | --- | --- | --- |
|  | n | % | n | % | n | % | *p*-value |
|  | 3,478 |  | 1,030 |  | 22,540 |  |  |
| **Age at delivery, years** |  |  |  |  |  |  | <.0001 |
| 20-34 | 2,509 | 72.1 | 651 | 63.2 | 17,684 | 78.5 |  |
| < 20 or ≥ 35 | 969 | 27.9 | 379 | 36.8 | 4,856 | 21.5 |  |
| **Parity** |  |  |  |  |  |  | <.0001 |
| no previous deliveries | 1,840 | 52.9 | 425 | 41.3 | 9,226 | 40.9 |  |
| 1 or more previous deliveries | 1,637 | 47.1 | 600 | 58.3 | 13,300 | 59.0 |  |
| unknown | 1 | 0.0 | 5 | 0.5 | 14 | 0.1 |  |
| **Pre-pregnancy BMI^a^** |  |  |  |  |  |  | <.0001 |
| < 18.5 | 126 | 3.6 | 3 | 0.3 | 661 | 2.9 |  |
| 18.5-24.9 | 1,510 | 43.4 | 201 | 19.5 | 11,569 | 51.3 |  |
| ≥ 25.0 | 1,629 | 46.8 | 255 | 24.8 | 6,429 | 28.5 |  |
| unknown | 213 | 6.1 | 571 | 55.4 | 3,881 | 17.2 |  |
| mean, (SD) | 26.40 | (6.16) | 27.07 | (5.88) | 24.48 | (4.87) |  |
| **Cohabitation** |  |  |  |  |  |  | <.0001 |
| married/co-habiting | 2,649 | 76.2 | 821 | 79.7 | 20,110 | 89.2 |  |
| single | 570 | 16.4 | 124 | 12.0 | 1,156 | 5.1 |  |
| unknown | 259 | 7.5 | 85 | 8.3 | 1,274 | 5.7 |  |
| **Smoking** |  |  |  |  |  |  | <.0001 |
| no | 1,921 | 55.2 | 590 | 57.3 | 18,584 | 82.5 |  |
| yes | 1,424 | 40.9 | 395 | 38.4 | 3,226 | 14.3 |  |
| unknown | 133 | 3.8 | 45 | 4.4 | 730 | 3.2 |  |
| **Socio-economic**  **status** ^b^ |  |  |  |  |  |  | <.0001 |
| upper white collar | 244 | 7.0 | 87 | 8.5 | 2,965 | 13.2 |  |
| lower white collar | 678 | 19.5 | 325 | 31.6 | 6,463 | 28.7 |  |
| blue collar | 357 | 10.3 | 162 | 15.7 | 2,455 | 10.9 |  |
| other | 728 | 20.9 | 249 | 24.2 | 3,129 | 13.9 |  |
| unknown | 1,471 | 42.3 | 207 | 20.1 | 7,528 | 33.4 |  |
| **Exposure to other psychiatric drugs**^c^ | 2,124 | 61.1 | 592 | 57.5 | 810 | 3.6 | <.0001 |
| **Maternal illness** |  |  |  |  |  |  |  |
| psychotic and other severe  mental disorders ^d^ | 1,344 | 38.6 | 452 | 43.9 | 70 | 0.3 | <.0001 |
| pre-gestational diabetes^e^ | 69 | 2.0 | 11 | 1.1 | 189 | 0.8 | <.0001 |
| gestational diabetes | 897 | 25.8 | 149 | 14.5 | 2,936 | 13.0 | <.0001 |
| other chronic  illness ^f^ | 195 | 5.6 | 51 | 5.0 | 1,353 | 6.0 | 0.2692 |

^a^ Data available from 2004/2005

^b^ The socioeconomic status was defined by maternal occupation at the time of delivery from Medical Birth Register. The categorization of occupational classes is based on the socioeconomic classification of Statistics Finland, a widely used classification of social position. We used the following categories: upper-white collar workers (administrative, managerial, professional and related occupations); lower-white collar-workers (administrative and clerical occupations); blue-collar workers (manual occupations) and others, including self-employed persons, students, unemployed, those at home with the child, and those with unknown occupation and socioeconomic status unknown. If education was recorded instead of occupation, these were transformed to socioeconomic status according to the level of education (<https://www.stat.fi/en/luokitukset/koulutusaste_oh/>).

^c^ Other psychiatric drugs, including anxiolytics (ATC codes N05B), hypnotics and sedatives (N05C), antidepressants (N06A), psycho stimulants (N06B), antidepressant-combination preparations (N06C), drugs used in addictive disorders (N07B)

^d^ From the Special Reimbursement Register

^e^Diagnoses (ICD-10) E10-E11 and O24.0-O24.1 or medical reimbursement for diabetes

^f^ One or more chronic illnesses (other than psychotic or severe mental disorder or pre-gestational diabetes) obtained from the Special reimbursement register (Kela).

**Supplementary Table S4.** Maternal characteristics tested as covariates by study outcome in imputed data.

| Covariate | Outcome; congenital major malformation | | | | | | | |
| --- | --- | --- | --- | --- | --- | --- | --- | --- |
|  | Any major malformation | Cardio-  vascular | Central  nervous system | Respiratory  tract | Orofacial cleft | GI | Urogenital | Musculoskeletal |
| ***P-value*** | | | | | | | | |
| Age at delivery | **<.0001** | **0.0519** | 0.1174 | **0.0644** | **<.0001** | **0.0254** | 0.8905 | **<.0001** |
| Parity | **<.0001** | **0.0268** | **0.0187** | 0.1788 | **0.0007** | **<.0001** | **0.0694** | **<.0001** |
| Pre-pregnancy BMI | **<.0001** | **<.0001** | **0.0004** | **<.0001** | **0.0007** | **<.0001** | **<.0001** | **<.0001** |
| Cohabitation | **<.0001** | **<.0001** | **<.0001** | **<.0001** | 0.8136 | **0.0944** | **<.0001** | **<.0001** |
| Smoking | **<.0001** | 0.1176 | **<.0001** | 0.7376 | **0.0288** | 0.2807 | 0.5263 | **<.0001** |
| SES | **<.0001** | **<.0001** | **0.0016** | **<.0001** | **<.0001** | **<.0001** | **<.0001** | **<.0001** |
| Other psychiatric drugs^1^ | **<.0001** | **<.0001** | **<.0001** | **0.0004** | **<.0001** | 0.5685 | **<.0001** | **<.0001** |
| Psychotic and other severe mental disorders | **<.0001** | **<.0001** | **<.0001** | **0.0806** | **<.0001** | **<.0001** | **<.0001** | **<.0001** |
| Pre-gestational diabetes | **<.0001** | **<.0001** | 0.1478 | **<.0001** | **<.0001** | **0.0403** | **<.0001** | **<.0001** |
| Gestational diabetes | **0.0002** | **0.0004** | **<.0001** | **<.0001** | **0.0083** | **0.0127** | **<.0001** | 0.1908 |

Analyses based on imputed data.

BMI; categorized body mass index, SES; socio-economic status, GI; gastrointestinal.

^1^ATC codes from following categories: N05B, N05C, N06A, N06B, N06C and N07B.
